# Natural overproduction of catalase by *Kocuria* sp. ASB 107: extraction and semi-purification

**Published:** 2017-12

**Authors:** Maryam Najari, Zahra Moosavi-Nejad, Elham Sadat Seyad Javad Javaheri, Ezat Asgarani

**Affiliations:** Department of Biotechnology, Faculty of Biological Sciences, Alzahra University, 1993893973, Tehran, Iran

**Keywords:** *Kokuria*, Catalase, Overproduction, Extraction, Semi-purification

## Abstract

**Background and Objectives:**

Because of importance of catalase in various industries, efforts have been made to find more suitable bacterial sources for catalase production. *Kocuria* is one of well-known catalase-producing genus. This is the first report about a new catalase-overproducing bacterial strain, *Kocuria* sp. ASB 107.

**Materials and Methods:**

*Kocuria* sp. ASB 107 had been isolated from Abe-Siah Spring in Ramsar in our previous report. The bacterial biomass freezed, thawed and then lysed by three different operations separately: ultrasound, lysing buffer and enzymatic digestion. The crude extract was subjected to ammonium sulfate precipitation (40 and 60% saturation). Quality and quantity of the semi-purification was checked by electrophoresis and measuring specific activity, respectively.

**Results:**

*Kocuria* sp. ASB 107 can be lysed by a freeze-thaw stage followed by lysozyme digestion and not by lysing buffer and not by ultrasound. Surprisingly specific activity of catalase in crude extract from *Kocuria* sp. ASB 107 was measured to be 195, 370 U/mg protein which is too much higher than other bacterial strains. The bacterium showed a relatively long growth curve about 40 hours. Semi-purification using ammonium sulfate precipitation was led in an increased specific activity up to about 7×10^6^ U/mg protein implying more than 3.6-fold purification.

**Conclusion:**

We have showed natural catalase-overproducing ability of *Kocuria* sp. ASB 107. Yield and purity of catalase from *Kocuria* sp. ASB 107 showed great potential in industrial application suggesting the strain as good source for mass production of catalase for treatment of H_2_O_2_-containing wastewater in comparison to other bacterial sources.

## INTRODUCTION

Catalase is an enzyme found in all aerobic organisms and micro-organisms. It catalyzes the decomposition of hydrogen peroxide to oxygen and water. As an anti-oxidative enzyme, catalase is a part of defense system which protects cells against the harmful effects of reactive oxygen species (ROS) such as superoxide anions, hydrogen peroxide and hydroxyl radicals (1–3).

On the other hand, hydrogen peroxide is increasingly used in industrial bleaching and sterilizing processes performed (4–6). Subsequently, it is necessary to apply an environmentally friendly process to remove the remaining hydrogen peroxide. For this reason, catalase is a good choice for application in dairy, textile, medicine and paper industries (7–10).

Efforts have been made to find more suitable bacterial sources for catalase production (11–13) especially for extremophiles or extremotolerant species (14–15) because of defensive role of catalase to decrease the raised concentration of hydrogen peroxide in extreme conditions. Not too long, it has been isolated a radio-resistant, psychrophilic *Kocuria* sp. ASB 107 from the Ab-e-Siah mineral radioactive spring (Ramsar, Mazandaran Province, Iran) (16). The bacterium has been characterized most closely related to *Kocuria rosea* DSM 20447T (99.7% sequence similarity) and *Kocuria polaris* DSM 14382T (99.5%) on the basis of morphological and biochemical characteristics plus 16S rRNA gene sequencing. Because of radioresistancy of *Kocuria* sp. ASB 107, it is logical to suppose its naturally high production of catalase and consequentially, good candidacy for catalase purification.

In this study, we have showed naturally catalase-overproducing ability of *Kocuria* sp. ASB 107. Moreover, we have investigated extraction, semi-purification and immobilization of its catalase.

## MATERIALS AND METHODS

### Bacterial strain and chemicals.

The strain *Kocuria* sp. ASB 107 had been isolated from Abe-Siah Spring in Ramsar (16). All chemicals were of analytical grade.

### Bacterial growth curve.

*Kocuria* sp. ASB 107 was initially cultured in Triptic Soy Agar (TSA) at 30°C for 48 hours. Bacteria of orange colonies were transferred to medium Tryptic soy broth (TSB) at 30°C and 150rpm on a rotary shaker. To following the bacteria growth, an aliquot of culture medium was picked up and its optical density was measured at 600nm.

### Kinetics of catalase production.

One milliliter of the TSB culture medium was harvested in fifth, fifteenth, twentieth, thirty-five hour of the bacterial growth and the precipitated cells were lysed using method C (see “catalase extraction”) and assayed for catalase activity as mentioned below (see “Enzyme assay”).

### Catalase extraction.

The bacterial suspension in 40^th^ hour of growth was centrifuged at 15,000g for 15 min at 4°C. The pellet was freezed (overnight at −20), thawed and lysed by three different operations separately:

- Physical method: The thawed biomass (0.2 g) was suspended in 1ml phosphate buffer (50mM, pH7) and lysed by sonication at 100% amplitude (1min on, 30 Sec off) for several cycles. The lysate was centrifuged at 15000g for 25 min at 4°C and the supernatant was used as crude cell extract.
- Chemical method: The thawed biomass (0.2 g) was suspended in 1 ml SET buffer (20% sucrose, 1.3 mM EDTA in Tris-HCl, 50mM, pH7) (17). After incubation for 10 min at 30°C, the resulted lysate was centrifuged at 15000g for 23 min at 4°C. The super-natant was used as crude cell extract.
- Enzymatic method: The thawed biomass (0.2 g) was suspended in 1ml phosphate buffer, 50mM, pH7 containing 0.5 mg lysozyme enzyme. After incubation for 80 min at 37°C, the lysate was centrifuged at 28000g for 20 min at 4°C. The supernatant was used as crude cell extract.

Catalase activity of all three cell extracts was assayed as mentioned below.

### Enzyme assay.

Catalase activity was measured spectrophotometrically by monitoring the rate of decrease in A_240_ using SHIMADZU UV-1800 spectrophotometer (18). The assay was initiated by adding enzyme solution (crude cell extract) to 35mM hydrogen peroxide in phosphate buffer, 50mM, pH7. The initial linear change in A_240_ was used to calculate the rate of hydrogen peroxide decomposition. The molar absorption coefficient for hydrogen peroxide at 240 nm was assumed to be 43.6 M^−1^.cm^−1^ (18). One unit of catalase activity is defined as the amount of enzyme required to convert 1μmol of H_2_O_2_ to water and oxygen per minute (19).

### Ammonium sulfate precipitation.

The crude extract was subjected to ammonium sulfate precipitation using information essentially offered in published procedure (20). All operations were carried out at 4°C. Solid ammonium sulfate was added to the pooled crude cell extract (resulted from method “C” of cell lysis) to reach 40% saturation of ammonium sulfate concentration. After 12 h incubation to complete precipitation, precipitated proteins were collected by centrifugation at 10800g for 20 min (pellet 40%). Second step of precipitation was performed by adding additional solid ammonium sulfate to the supernatant to reach 60% ammonium sulfate concentration. After 12 h incubation, the new precipitated proteins were collected by centrifugation at 17300g for 20 min (pellet 60%). The two pellets were dissolved in a small amount of phosphate buffer 50mM, pH7.0. The dissolved pellets as well as the supernatant of fraction 60% were dialyzed against the same buffer (10 kDa cutoff). Dialysates were used to measured catalase activity as well as protein content as mentioned below.

### Protein assay.

Protein concentration was determined using Bradford method with bovine serum albumin as the standard (22).

### Denaturing electrophoresis (SDS-PAGE).

Electrophoretic pattern of proteins in crude cell extract and partially purified catalase were determined by sodium dodecyl sulfate-polyacrylamide gel electrophoresis (SDS-PAGE) on a 12% acrylamide gel in tris-HCl buffer, 50 mM, pH 8.3 with constant voltage of 100V, according to the method of Laemmli (23).

### Statistical analysis.

In order to determine the measurements reproducibility, all measurements were performed in triplicate. Results are presented as mean values ±SD. RSD values were lower than 10% for all measurements. P-values lower than 0.01 was assumed as statistical difference between experimental points. Data analysis was done using Microsoft Excel 2016.

## RESULTS

**Fig. 1.**
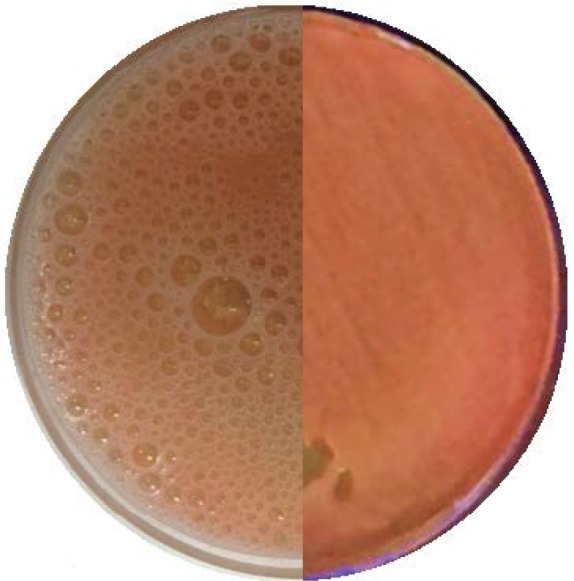
Oxygen bubbles produced by *Kocuria* sp. ASB 107 in TSA culture after catalase test

Catalase activity of the isolated strain, *Kocuria* sp. ASB 107 was showed by pouring hydrogen peroxide (3%) on a TSA culture of the bacterium and producing a lot of bubbles (Fig. 1).

To confirm the fact that catalase is an intracellular enzyme, catalase test was performed for both super-natant and pellet of a centrifuged-cell suspension. No considerable activity was observed in supernatant, while the pellet showed an intense positive result for catalase test.

**Fig. 2.**
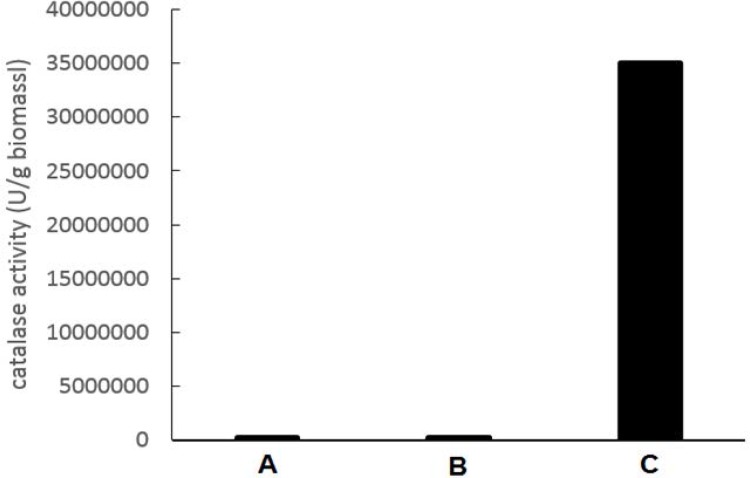
catalase activity in lyset resulting from three diffrent lysis methods: A) physical method (ultrasound), B) chemical method (SET buffer), C) biologycal method (lysozyme enzyme)

Bacterial cells in the pellet were lysed using three methods (see “Materials and Methods”). As shown in Fig. 2 the lysate obtained from physical method (ultrasound) and chemical method (SET Buffer) did not have considerable catalase activity, but on the contrary, lysate obtained from lysozyme method had about 35×10^6^ unit catalase activity in one gram of wet biomass. Specific activity of crude extract was 195, 370 U/mg protein. The enzymatic method of lysis was used in the next experiments.

**Fig. 3.**
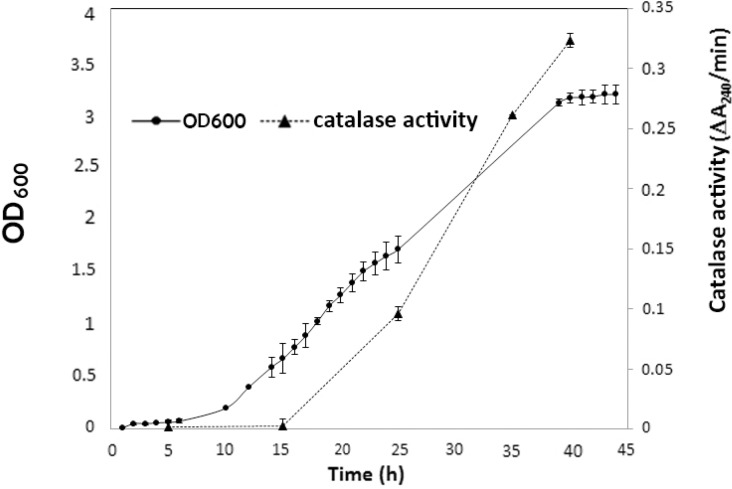
Time course of growth and catalase production by *Kocuria* sp. ASB 107 in TSB

To determine optimal time of catalase production in *Kocuria* sp. ASB 107, bacterial growth curve was plotted in TSB medium culture (Fig. 3). Accordingly, the bacterium had a long growth curve (about 2 days) with a 10-hour lag phase, a 29-hours log phase and a late stationary phase at 39^th^ hour of culture. Meanwhile catalase production started in the middle of log phase, showed a fast rising rate at the late log phase and maximum amount after about 40 h of cultivation at stationary phase.

**Fig. 4:**
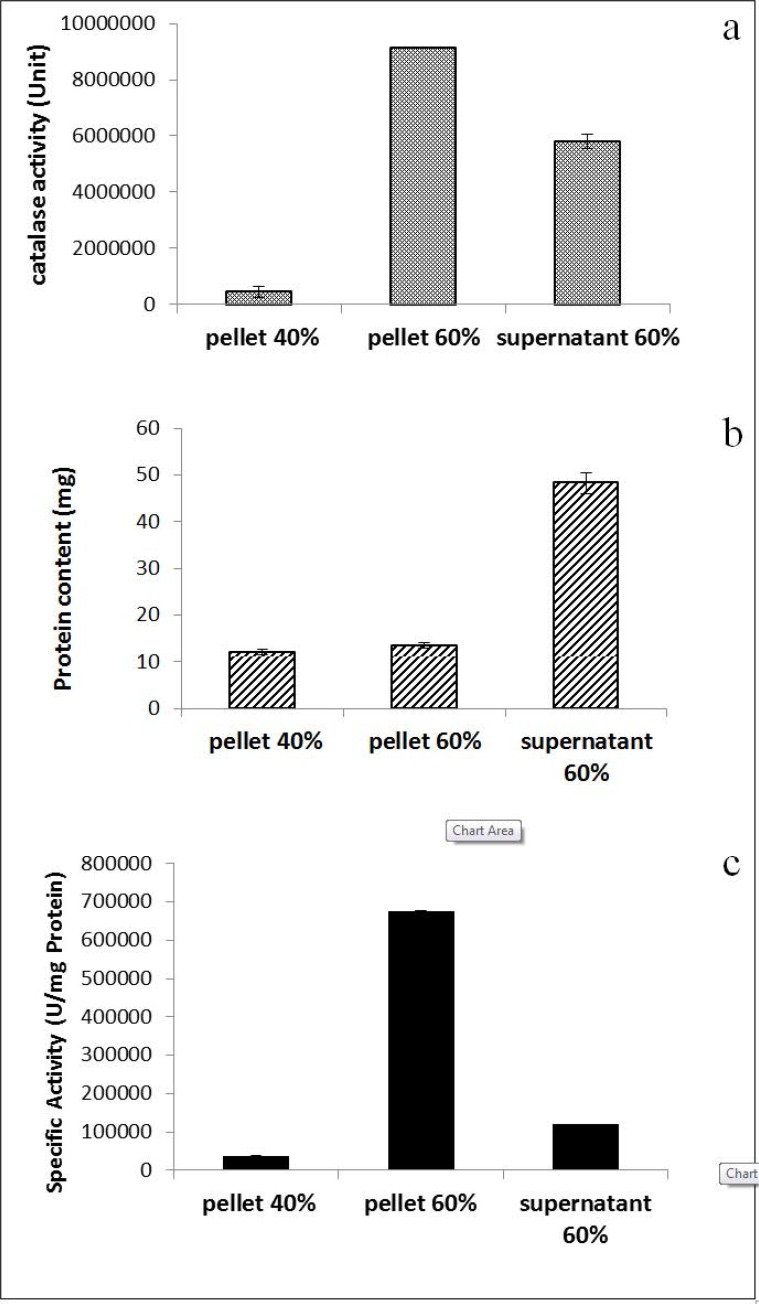
Catalase activity (a), protein content (b) and specific activity of the three ammonium sulfate fractions resulted from semi-purification procedure.

Catalase was semi-purified from *Kocuria* sp. ASB 107 at steady state of growth curve (40^th^ hour) showing maximum catalase production. The semi-purification was performed by ammonium sulfate precipitation method which is a common and important method as offered by other researchers (23 and 24). In Fig. 4, catalase activity (Fig. 4a), protein content (Fig. 4b) and specific activity (Fig. 4c) of the three resulted fractions have been showed. Pellet 60% had maximum activity in comparison to other fractions. Although supernatant of 60% also showed a relatively considerable amount of catalase (Fig. 4a) but it contained high amount of proteins (Fig. 4b) as its specific activity became more than 6 fold less than pellet 60% (Fig. 4c) indicating the existence of a lot of impurity in supernatant of 60%. As was found in figure 4c, one g of protein in pellet 60% was contained of 6.9×10^5^ units of catalase.

**Fig. 5:**
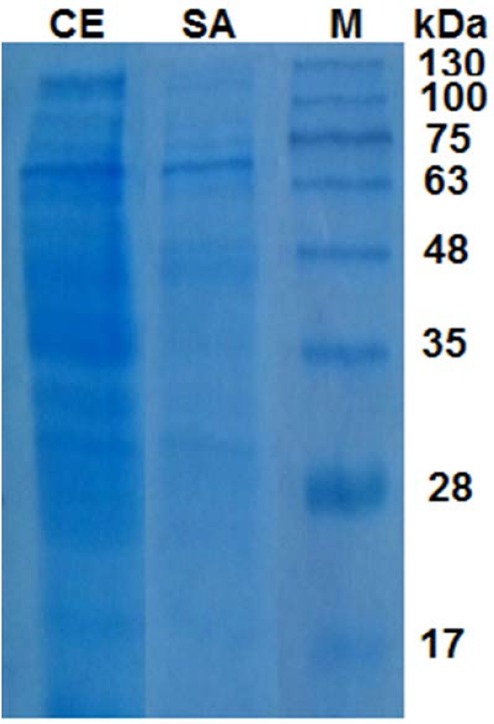
SDS-PAGE of crude extract (CE) and 60% ammonium sulfate fraction (SA) from *Kocuria* sp. ASB 107 and molecular weight marker (M).

Electrophoresis under denaturing conditions (SDS-PAGE) revealed that many protein impurities had been removed from crude extract by 60% saturation of ammonium sulfate (Fig. 5).

## DISCUSSION

Because of importance of catalase in various industries (7–10), efforts have been made to find more suitable bacterial sources for catalase production (25–29). *Kocuria* is one of well-known genus which produces catalase to defense against environmental oxidative stress (30–33). *Kocuria* sp. ASB 107 is new member of the genus which has been isolated from Ab-e-Siah mineral radioactive spring in north of Iran (16). It is resistant to several stresses such as ultraviolet ionizing radiation (16). Having a considerable amount of catalase (Fig. 1) is experimentally related to resistance to oxidative stress caused by ionizing radiation (34 and 35). According to our observations, *Kocuria* sp. ASB 107 can be lysed by a freeze-thaw stage followed by lysozyme digestion (Fig. 2).

Surprisingly one gram of wet biomass was contained too much amount of catalase (about 35×10^6^ units equal to 195,370 U/mg protein). The natural overproduction of catalase by *Kocuria* sp. ASB 107 was rechecked several times for certainty. As the first report, such a high specific activity of crude extract is important because it has not exceeded 40,526 U/mg protein in other microorganisms. The specific activity of crude extracts of several main catalase-producing bacterial strains has been compared in Table 1. The specific activity of non-purified catalase from *Kocuria* sp. ASB 107 is considerably higher than other reports even in comparison to purified recombinant catalase (36).

**Table 1.** Comparison of catalase specific activity of crude extracts from several bacterial strains

**Bacterium**	**Specific activity (U/mg protein)**	**Reference**
*Acinetobacter sp*. YS0810	746	12
*Vibrio rumoiensis S-1T*	7,300	14
*Rhizobium radiobacter* Strain 2-1	30,420	37
Recombinant *E. coli* BL21 (DE3)	40,526*	36
*Kocuria* Asp. SB 107	195,370	This study

* specific activity of purified catalase.

Despite of some other strain (37), *Kocuria* sp. ASB 107 showed a relatively long growth curve, while the production of catalase varied depending on growth phase (Fig. 3): catalase production started very late 5 hours after starting exponential phase but a substantial increase in catalase activity was observed in the middle of stationary phase. This pattern is very similar to *Rhizobium radiobacter* Strain 2-1 (37) and different from *Deinococcus radiophilus* which showed a gradual increase in total catalase activity during exponential phase (35).

Semi-purification using ammonium sulfate precipitation was led in an increased specific activity up to about 7×10^6^ U/mg protein (Fig. 4c) implying more than 3.6-fold purification which is more than similar report (38). This occurred in precipitant of 60% ammonium sulfate. Although protein content of supernatant of 60% was 4-fold higher than protein content of its precipitant (Fig. 4b) but measuring their catalase activity implies that most of proteins in supernatant of 60% are impurities (Fig. 4a). The electrophoretic pattern also confirmed this deducing (Fig. 5).

In summary, this is the first report about a new catalase-overproducing bacterial strain, *Kocuria* sp. ASB 107. Yield and purity of catalase from *Kocuria* sp. ASB 107 showed great potential in industrial application suggesting this strain is good source for mass production of catalase for treatment of H_2_O_2_-containing wastewater in comparison to other bacterial sources. More experiments are necessary in order to full purification and characterization of catalase from *Kocuria* sp. ASB 107 as a naturally catalase-overproducing bacterial strain.
